# Percutaneous Cryoablation of Metastatic Lesions from Colorectal Cancer: Efficacy and Feasibility with Survival and Cost-Effectiveness Observations

**DOI:** 10.5402/2012/942364

**Published:** 2012

**Authors:** Hyun J. Bang, Peter J. Littrup, Brandt P. Currier, Dylan J. Goodrich, Minsig Choi, Lance K. Heilbrun, Allen C. Goodman

**Affiliations:** 1 Wayne State University, Detroit, MI 48201, USA; 2 Department of Radiology, Wayne State University, 22473 Milner Street, St. Clair Shores, MI 48081, USA; 3 Karmanos Cancer Center, Detroit, MI 48201, USA

## Abstract

**Purpose:**

To assess feasibility, complications, local tumor recurrences, overall survival (OS) and estimates of cost-effectiveness for multi-site cryoablation (MCA) of oligo-metastatic colorectal cancer (mCRC) in a prospective study.

**Materials and Methods:**

111 CT and/or US-guided percutaneous MCA procedures were performed on 151 tumors in 59 oligo mCRC patients. Mean patient age was 63 years (range 21–92 years), consisting of 29 males and 30 females. Tumor location was grouped according to common metastatic sites. Median OS was determined using the Kaplan-Meier. Estimates of MCA costs per LYG were compared to historical values for systemic therapies.

**Results:**

A mean 1.9 MCAs per patient were performed with a median clinical follow-up of 12 months. Major complication and local recurrence rates were 8% (9/111) and 12% (18/151), respectively. Median overall-survival (OS) was 23.6 months with an estimated 3-year survival rate of ~30%. Cryoablation remained cost effective with or without the presence of systemic therapies, with an adjunctive cost-effectiveness ratio (ACER) of $39,661–$85,580 per LYG.

**Conclusions:**

Multi-site cryoablation had very low complication and local recurrence rates, and was able to provide local control even for diverse soft tissue locations. Even as an adjunct to systemic therapies, MCA appeared cost-effective, with apparent increased survival.

## 1. Introduction

Colorectal cancer (CRC) is the 3rd most commonly diagnosed cancer and the 2nd most common cause of death among cancers in adults [1]. An estimated 141,210 new cases of CRC were diagnosed in the United States in 2011, producing 49,380 fatalities [2]. Metastatic CRC (mCRC) develops in 50–60% of patients (50,000–60,000), is usually incurable, and has an overall 5-year survival rate of 11.6% [3, 4]. Of patients diagnosed with CRC, 50% develop liver metastases, while 10–15% will eventually develop lung metastases [5].

Recent advancements using various chemotherapeutic agents have broadened the options available to mCRC patients, particularly when coupled with targeted treatments (e.g., monoclonal antibodies). 5-fluorouracil monotherapy (5-FU) alone or in combination with other systemic therapies continues to be a standard treatment and has achieved median survivals ranging from 8.5 to 15.4 months [6–8]. With the addition of the monoclonal antibody bevacizumab to these treatments, survival may be able to be extended to 21.5 months [9].

Hepatic resection and metastasectomy provide the best prognoses for mCRC patients [10, 11]. However, survival rates for hepatectomy patients may be more attributable to common exclusion criteria: (1) lack of resection of primary tumor, (2) lymph node positive primary tumor, (3) extrahepatic disease, (4) CEA level > 200 U/mL, and (5) largest tumor > 5 cm. Three or more of these criteria indicate high risk, and only an estimated 15% of mCRC patients remain eligible for surgery [12, 13]. A significant portion of patients with mCRC are thus unable to experience the survival benefits of surgery and must resort to systemic treatments.

Minimizing procedure-related morbidity and treatment costs while still producing survival benefits are important for the adoption of minimally invasive treatments targeting oligometastatic disease [14]. Minimally invasive percutaneous ablation techniques are available to a broader selection of mCRC patients (compared to surgical excision) and are effective when paired with chemotherapy and/or targeted treatment regimens [15, 16]. Radiofrequency ablation (RFA) is more widely used for pulmonary and hepatic tumor ablations but may be more limited than cryoablation for tumors in diverse soft tissue locations [17], or those abutting crucial structures. Cryoablation has also been used in the local control of tumors in the liver and lungs [18, 19], but the benefits of multisite cryoablation (MCA) for oligometastatic CRC (oligo-mCRC) lesions are relatively unstudied. The visible treatment zone of cryoablation, lower pain, and minimal morbidity allowed us to apply our established cryoablation techniques [19–26] to many metastatic sites for local control of mCRC.

The purpose of this study was to assess the potential role of multisite cryoablation of oligometastatic CRC by evaluating complications, recurrences, overall survival (OS), and projected costs as an adjunctive treatment for mCRC. Preliminary cost-effectiveness estimates for MCA were compared to historical values for common systemic treatments [6–9] to assess the potential economic impact of MCA.

## 2. Materials and Methods

### 2.1. Patients

Consecutive patients with oligo-mCRC read and signed an authorization form issued under the Health and Insurance Portability and Accountability Act of 1996 (HIPAA). All patients also signed a separate consent form detailing the procedure, as well as an investigational review board approved consent form for prospective collection of procedure, imaging, and clinical data. Mean patient age was 63 years (range 21 to 92 years) at time of first procedure. Patient group consisted of 29 males and 30 females. The six procedural locations included *liver*, *lung*, and 4 soft tissue sites: *retroperitoneal*, *superficial*, *intraperitoneal*, and *bone* (Table 1).

The patients included in this study were retrospectively confirmed as mCRC through thorough review of their patient charts, pathology reports, imaging findings, and correlation with PET-positive lesions. Patient charts were evaluated by a gastrointestinal oncologist (MC) who noted whether patients received best supportive care (BSC) or any chemotargeted therapy regimen before or after MCA. For comparison in our cost evaluations, these regimens included 5-flourouracil monotherapy (5-FU); 5-FU with leucovorin; 5-FU with oxaliplatin and leucovorin (FOLFOX); 5-FU with irinotecan and leucovorin (FOLFIRI); FOLFIRI with bevacizumab; and cetuximab with irinotecan.

### 2.2. Inclusion Criteria

Inclusion criteria for cryoablation consisted of a localized soft tissue mass <7 cm that was biopsy proven, deemed suspicious from a CT showing an enhancing, growing mass, or a positive PET scan. Patients typically had less than 5 cancerous foci per organ site to avoid compromising safety in patients with advanced disease; MCA was only conducted if it was believed all metastases present at the time of first procedure could be ablated over the course of one or multiple procedures, and patients were generally referred by oncologists or surgeons for local control of oligo-mCRC. Tumors in multiple locations were done in single- or multiple-staged cryoablation procedures according to projected feasibility and/or safety. MCA also treated additional foci developing over time in subsequent procedures. All cases were reviewed and performed by a single radiologist with 20 years of interventional and cross-sectional imaging experience (PJL). Ablation was conducted within the context of providing thorough ablation for oligo-mCRC patients and a chance at providing active disease treatment comparable to surgical metastasectomy.

### 2.3. Cryoablation Procedure

The primary goal for each cryoablation procedure was to achieve sufficient probe distribution (e.g., ~1 cryoprobe for each cm tumor diameter) to reach cytotoxic temperatures less than −20°C covering all tumor margins. Probe type (i.e., 1.7 or 2.4 mm outer diameter) and number were recorded for each ablation site. Cryoablation planning techniques/procedural details and associated hydrodissection protection measures for renal, pulmonary, soft tissue, and breast tumors have been previously described [22–26].

### 2.4. Imaging and Followup

Real-time ultrasound (US) (Logiq 700; GE Medical Systems, Milwaukee, WI) was solely used to place and monitor cryoprobes during procedures in all superficial locations, which consisted of predominantly subcutaneous, muscular, and/or lymph node metastases within the extremities or torso wall. Computed tomography (CT) was used as the primary imaging modality for planning, procedure guidance, and treatment followup in the remaining 5 procedural sites (Table 1). MR imaging was used as needed for improved tissue/tumor discrimination or iodine allergies. Tumors were measured in three dimensions, noted on axial images in their greatest transverse and anteroposterior extent, and craniocaudal measurements were obtained via sagittal and/or coronal reconstructions. In followup, enhanced CT or MRI images were obtained at 1, 3, 6, 12, 18, and 24 months and yearly thereafter as available. The overall ablation zone was similarly measured in three dimensions.

### 2.5. Complications

All treatment-related complications were categorized in accordance with the Common Terminology Criteria for Adverse Events Version 3.0 (CTCAE v3) of the National Cancer Institute, similar to prior cryoablation series [22, 24]. Complications were not linked to cost estimates. A formal decision analysis model was not yet considered appropriate for our initial cost-effectiveness estimates.

### 2.6. Recurrences

The ideal goal of cryoablation is to achieve complete ablation of a tumor focus with minimal damage to surrounding soft tissues. However tumor recurrence may occur at the site of cryoablation. Local recurrences were separated into *procedural* and *satellite* etiology and do not address additional metastatic disease since patients were stage IV and treated for palliation. A *procedure*-related recurrence was defined as any recurrence within the ablation zone resulting from an inadequate, sublethal isotherm likely along the tumor rim (positive margins) [27]. *Satellite* lesions were located less than 1 cm beyond the ablation zone likely resulting from adjacent microscopic foci of the tumor.

### 2.7. Survival

Overall survival for patients undergoing MCA was determined using the Kaplan-Meier (K-M) estimator in the Lifetest procedure in SAS 9.2 software (SAS Institute, Inc., Cary, NC). Our patients had often failed other therapies yet were selected for limited remaining oligo-mCRC lesions. OS was measured from the time of the first MCA procedure until death or until the most recent followup for vital status determination. Due to modest sample sizes (or numbers of events), OS statistics (e.g., median, 1-year rate, etc.) were estimated more conservatively using linear interpolation between successive event times on the K-M curve [28]. All point estimates of OS statistics were accompanied by a 95% confidence interval (CI).

### 2.8. Cost

We explored inflated cost estimates for MCA to gain insight whether the palliative use of MCA had reasonable potential for future more detailed cost-effectiveness analyses. Our cost estimates also contain billing charges, rather than estimates of direct and/or indirect costs [29]. These cost estimates served as a potential economic counterbalance to any survival benefit noted for MCA, especially since ablation may be perceived as only adding costs to a palliative disease state.

A total cost of $11,000 per cryoablation procedure represents a high-end estimate from mean professional fees ($2,000), disposable equipment fees ($4,000 for 3 cryoprobes), and hospital fees ($5,000). Average cost of more frequent follow-up imaging examinations of $42,000 encompassed 6 follow-up CT imaging sessions at $7,000/CT (e.g., 1, 3, 6, 12, 18, and 24 months and yearly thereafter). This high value for each CT session reflected our institution’s 2010 Medicare technical component guidelines of $2,171, $2,396, and $1,390 for chest, abdomen, and pelvic CT, respectively, and professional fees of approximately $350/scan. In the event that MR was preferred or deemed more appropriate for that patient or tumor location, no significant cost difference was assumed based on our 2010 Medicare guideline of $2,171 for each MR exam per anatomic site. The mean number of procedures per patient was used to determine the cost per patient. However, the overlapping schedule in follow-up imaging after the second ablation did not justify counting follow-up imaging charges more than once, such that only the total ablation charge was multiplied by the number of ablations per patient.

Additionally, patients in this study may have had chemo-targeted treatments at some point. Costs of MCA were thus also considered in an *adjunctive* role and *added* to each therapy comparison, then divided by the overall LYG for MCA in this study. We termed this approach an adjunctive cost-effectiveness ratio (ACER) to more accurately estimate scenarios encountered by our patients. ACERs below $100,000 per LYG were considered cost-effective [29].

## 3. Results

### 3.1. Patients, Procedure, and Followup

A total of 59 patients underwent 111 procedures on 151 tumors. A detailed breakdown of the average tumor sizes and location can be found on Table 1. The cryoablation zone was well defined by CT as a hypodense ice ball with a mean ablation diameter of 5.5 cm generated by a mean 3.47 probes, while mean tumor diameter was 3.7 cm. Average patient age was 63 at time of first procedure (range 21–92 years). The mean follow-up time for all patients was 12 months. The following percentages of patients were exposed to treatment before or after MCA, respectively: 7% (4/59): 3% (2/59) 5-FU mono-therapy; 8% (5/59): 0% (0/59) 5-FU with leucovorin; 15% (9/59): 7% (4/59) 5-FU with leucovorin and oxaliplatin (FOLFOX); 8% (5/59): 5% (3/59) 5-FU with leucovorin and irinotecan (FOLFIRI); 12% (7/59): 5% (3/59) FOLFIRI with bevacizumab; 3% (2/59): 5% (3/59) cetuximab with irinotecan; and 53% (31/59): 32% (19/59) other. A total of 80% (47/59) of our patients received chemotargeted therapy at some point. Although 75% (44/59) of patients were administered systemic therapy before MCA, 58% (34/59) of patients were not given any chemotargeted regimen following their first MCA procedure.

### 3.2. Complications

Table 2 outlines the complications based upon severity and tumor location. Overall, nine procedures (8%) resulted in a grade 3 complication or worse. A grade 4 hemothorax occurred during ablation of a right hepatic dome metastatic lesion, in which the ablation zone reached 10 cm. The patient had a small retroperitoneal hemorrhage and moderate-sized right pleural effusion likely related to hemorrhagic products. In response to the patient’s blood pressure dropping to 80/40, anesthesia staff began intravenous fluid resuscitation. Selective angiography of the right 11th intercostal artery demonstrated a bleed which was then embolized.

A grade 4 hematoma/active bleeding occurred in a 66-year-old patient following cryoablation of a 6.5 cm posterior right hepatic mass involving an ~10 cm ablation zone. The patient was resuscitated with two liters of normal saline before admission to critical care, where he received four units of fresh frozen plasma, five units of platelets, and two units of red blood cells. Patient remained in the ICU for three days before being discharged.

The final grade 4 complication occurred in a 78-year-old patient whose platelet count dropped as low as 8,000, with a parallel drop in hemoglobin following cryoablation of a 3.6 cm liver mass. The ablation zone in this procedure reached up to 7 cm. Patient remained in recovery for a total of eight days before platelets returned to 20,000 and appeared to be continually rising.

A single death occurred (grade 5 complication) in a 51-year-old patient after treatment of 3 liver lesions, the largest measuring 4.3 cm, with the greatest ablation diameter being 8 cm. Patient initially received solumedrol 100 mg IV as a stress dose but was discontinued on the floor due to initial stable blood values. Over the next few days the patient experienced tachycardia, respiratory distress, hypotension, and a hemothorax, likely related to delayed thrombocytopenia. The patient became anuric and progressively acidotic before experiencing multisystem organ failure five days after the procedure.

### 3.3. Recurrences

A breakdown of local tumor recurrences and their classification as procedural or satellite etiology is shown in Table 3. Overall, procedures resulted in 4 (3%) procedure-related recurrences and 14 (9%) satellite recurrences were noted. One recurrence was seen in the soft tissue cohort. Three of the procedural recurrences occurred in one patient on a single tumor that was abutting the hilum. Subsequent ablations were successfully performed on 11 of the 18 total recurrences (4 procedural, 7 satellite), meaning these lesions showed no further local recurrence. Therefore, the overall ablation effectiveness rate was observed to be 95% (144/151).

### 3.4. Survival

The Kaplan-Meier survival curve is shown in Figure 4. The median survival time from patients undergoing cryoablation procedures was 23.6 months from the time of the procedure. Projected three-year survival rate was determined to be ~30% for our MCA patients. Figure 5 displays the median survival for patients who received either chemotargeted therapy after MCA versus those who only received best supportive care. The chemotargeted group displayed a median survival of 24.8 months with a 3-year OS rate of 33%, while the BSC group had a median survival of 23.5 months and a 3-year OS rate of 22%.

### 3.5. Cost

In all cases, “upper bound” cost estimates produced total cost of each cryoablation procedure and frequent imaging followup of $53,000 ($11,000/procedure plus $42,000 total for imaging followup). Multiple metastatic lesions were treated in an average of 1.9 procedures per patient, making the estimated upper cost per patient of $73,900 (i.e., $11,000 * 1.9 + $53,000).

Table 4 demonstrates our adjunctive cost-effectiveness (ACER) evaluations for MCA based on comparisons with established values from current literature for six mCRC therapies: 5-flourouracil monotherapy (5-FU); 5-FU with leucovorin; 5-FU with oxaliplatin and leucovorin (FOLFOX); 5-FU with irinotecan and leucovorin (FOLFIRI); FOLFIRI with bevacizumab; and cetuximab with irinotecan [6–9]. Our MCA estimate of cost per total LYG of $37,543 (i.e., $73,900/1.97) appears encouraging for future detailed analysis, especially since the ACER for MCA was cost-effective versus all chemotargeted therapy protocols, with the average being $62,517 per LYG.

## 4. Discussion

This study suggests feasibility, safety, and potential cost-effectiveness of MCA for patients with oligo-mCRC. We will first summarize our findings and then cover their individual implications. Other than an unusual death following a large hepatic cryoablation, perhaps related to an atypical and/or delayed cryoshock phenomenon, mortality and procedural morbidity associated with MCA were minimal. Local tumor recurrence and overall morbidity for this study were low and did not appear to be dependent upon tumor location. Our projected three-year survival of ~30% in our MCA patients approaches the encouraging rates noted for RFA in hepatic tumors originating from mCRC [15, 16]. A total of 80% of our patients received some form of chemotherapy regimen prior to MCA. However, only 42% of patients were administered systemic therapy after their first MCA procedure, suggesting local control of oligometastases may reduce the need for additional regimens. Our preliminary cost estimates suggest that MCA remained cost-effective even when added to the cost of chemotargeted regimens.

Although our survival rates could have been achieved by RFA or microwave ablation, MCA may be a viable treatment alternative for oligo-mCRC patients. For instance, the high impedance in the lung may limit the procedural effectiveness of RFA, as this represents a location with low electrical resistance, making the ablation zone difficult to predict (Figure 2). In a study including 55 nonsurgical patients receiving RFA for the treatment of pulmonary metastases, a local recurrence rate of 38% was noted [30], compared with our study which elicited a local recurrence rate of 18% (6/33) (Table 3). In conjunction with broad inclusion criteria, cryoablation exhibited unique aspects that may offer a favorable treatment option for patients limited isolated metastases, involving most organ and soft tissue sites. By visualizing the ablation zone’s advancement beyond all tumor margins, we were able to consistently achieve adequate ablation. In cases where inadequate ablation was speculated, probes could be added or repositioned to cover the region of suspect in the second freeze cycle.

**Figure 1:**
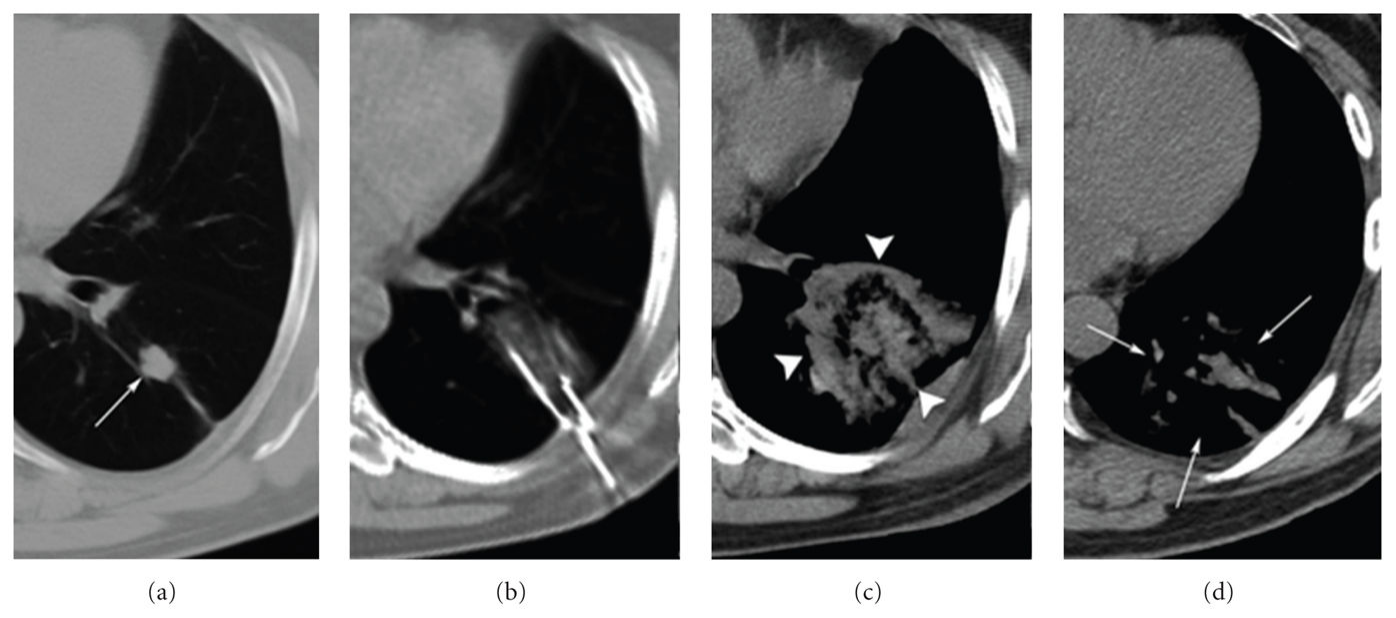
61-year-old male with metastatic colon cancer status after multiple RF and hepatic cryoablations as well as prior pulmonary cryoablations due to refusal to consider systemic chemotherapy presents for cryoablation of a growing satellite focus in the left lung at a previous ablation site. Axial CT images (from left to right) demonstrate the growing satellite focus (single arrow) measuring 1.8×1.8×1.5 cm which was thoroughly ablated using three cryoprobes in a triple freeze cycle. The ablation zone measured 5.4 × 4.3 × 4.5 cm which later resorbed to a nonenhancing ablation site measuring 3.9 × 2.5 × 2.5 cm.

**Figure 2:**
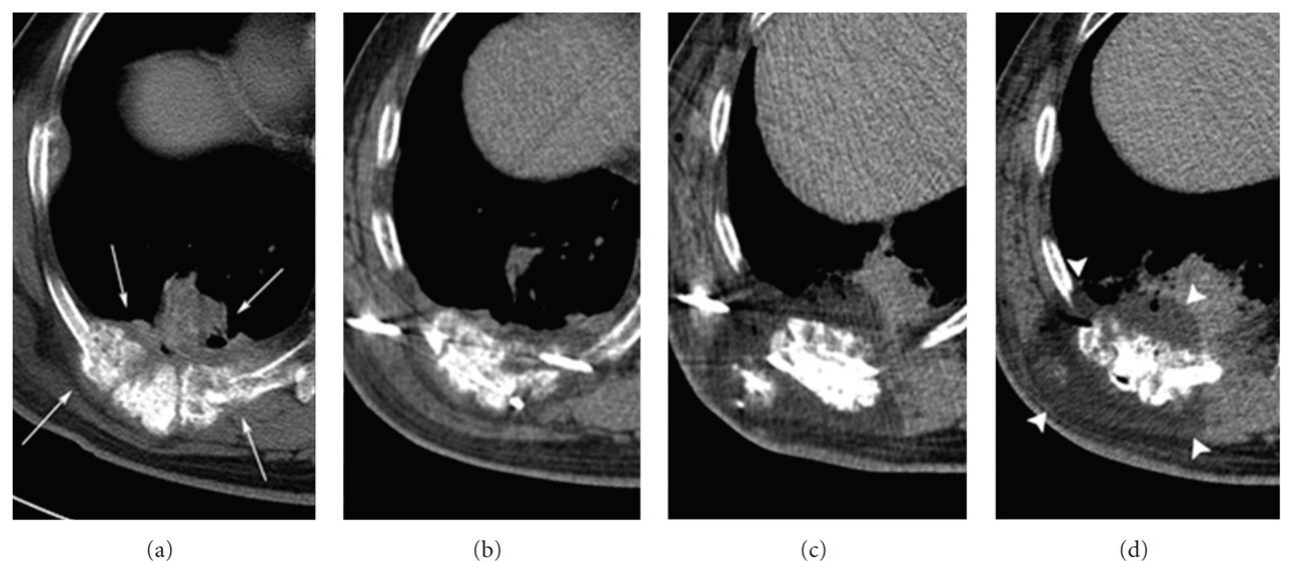
64-year-old male with metastatic colon cancer with prior RF ablation of a hepatic lesion presents with chronic pain from 4 right posterior chest wall masses involving the ribs, pleura, and adjacent musculature containing diffuse calcifications. Axial CT images (from left to right) demonstrate the total area of these 4 abutting masses to measure approximately 10 × 6.5 × 10 cm (a). A total of eight 2.4 mm cryoprobes were utilized during the procedure, with seven probes initially placed for the first freeze cycle, and an additional eighth probe was placed to cover the superficial/lateral tumor margin in the second freeze cycle ((b) and (c)). Up to 60 cc of saline were continuously injected to protect the overlying skin from the ablation zone. Final ice formation appeared to cover all tumor margins and measured 12 × 8 × 12 cm (d).

**Figure 3:**
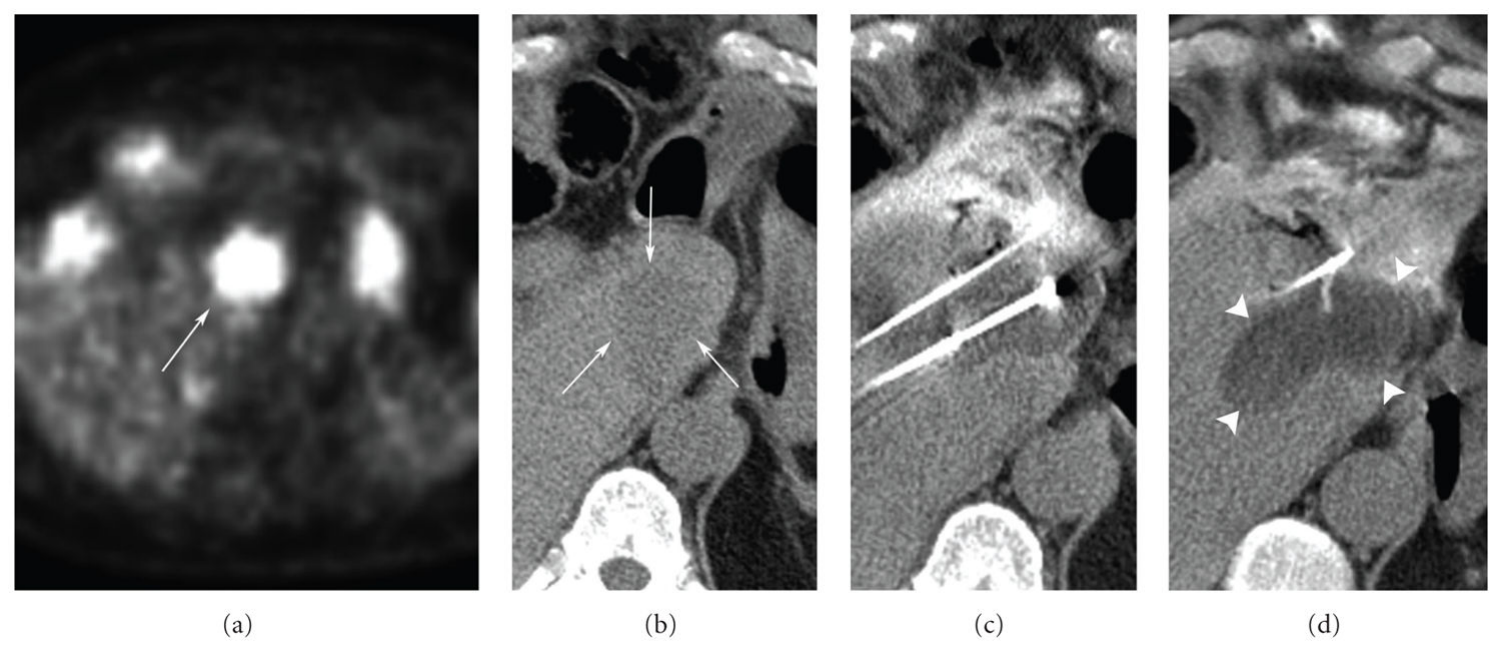
76-year-old male presenting with an FDG-PET positive recurrent lesion (a) from a cryoablation procedure 5 months prior. The caudate mass lies just anterior to the IVC and measures 3.3 × 3 × 3 cm (b). In order to avoid damaging the adjacent bowel, an 18-gauge Trocar needle was placed along the anterior/superior margin of the tumor, allowing for the injection of saline to provide hydrodissection protection. A total of three 2.4 mm cryoprobes bracketed the tumor, two of which abutted the IVC. Following a two, ten-minute freeze cycles ((c) and (d)), the ablation zone was visualized to extend beyond all tumor margins and produced final ice measurements of 4.2 × 5.5 × 5 cm. No new local recurrence was noted in subsequent follow-up imaging.

**Figure 4:**
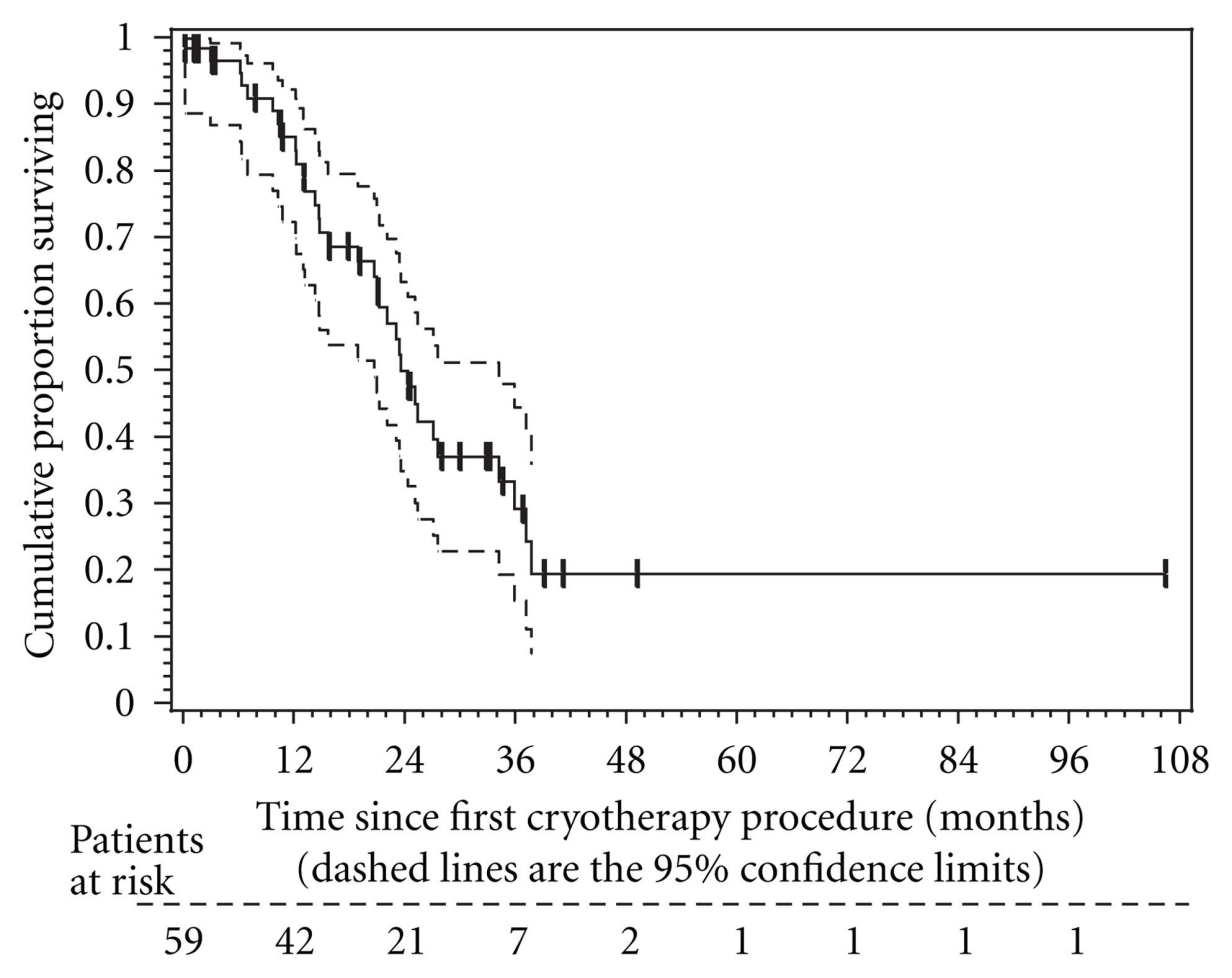
The Kaplan-Meier estimate of overall survival (OS) in the 59 study eligible patients. The dashed lines represent the 95% confidence interval (CI) about each successive estimate of the survival rate. The median OS was 23.6 months (95% CI, 20.7–34.2 months). The 2-year OS rate was 49% (95% CI, 34–63%). The 3-year OS rate was 33% (95% CI, 9–56%).

**Figure 5:**
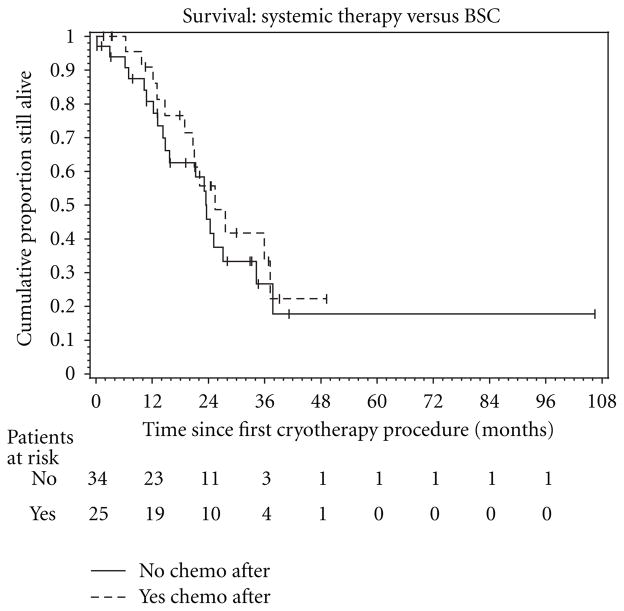
The Kaplan-Meier estimate of overall survival (OS) in the 59 study eligible patients. The dashed lines represent the 95% confidence interval (CI) about each successive estimate of the survival rate. The median OS was 24.8 months (95% CI, 18.3–36.5 months) for patients who received chemotargeted therapy following their first MCA procedure, and 23.5 months (95% CI, 14.2–34.1 months) for patients who only received best supportive care following first MCA. The 2- and 3-year OS rate for the chemo-targeted group was 52% (95% CI, 29%–75%) and 33% (95% CI, 9%–56%), respectively. For the BSC only group, the 2- and 3-year OS rate was 44% (95% CI, 25%–63%) and 22% (95% CI, 3%–41%), respectively.

An additional characteristic of cryoablation relates to conservation of vessel structural integrity throughout the freezing process (Figure 3). For centrally located pulmonary metastases, cryoprobes can be placed closely to mediastinal and hilar vessels under CT guidance to negate the thermal exchange occurring between vasculature and the ablation zone without fear of damaging vessel or bronchial architecture [19]. For instance, the patient depicted in Figure 1 presented with a tumor which had recurred after multiple RFA treatments and two cryoablation procedures, who was unwilling to receive further chemotargeted therapy. The presence of major vasculature in close proximity to this lesion presented challenges to both procedural success as well as complication susceptibility. Nevertheless, following the third cryoablation procedure targeting this lesion, no local recurrence was observed, and the procedure was well tolerated. When multiple ablations were conducted in our patient group on recurrent tumors of the lung and liver, overall ablation effectiveness was observed to be 95%. The efficacy of MCA appears to be similar to the feasibility of the procedure, with a major complication rate of 8% observed in our study.

Relative improvements in OS are measured in LYG but should also assess relative morbidities encountered by those treatments, especially when the associated prolongation of OS is relatively short. Adjustments to LYG usually account for time spent in high functioning states, or quality-adjusted life years (QALY), but require detailed quality-of-life instruments and/or surveys. Such analysis was beyond the scope of the study, and we therefore acknowledge our LYG estimates for MCA may be better considered as an adjunct to systemic treatments. Similarly, using overall/3-year survival as the principal statistic appears acceptable in current literature evaluating RF [15, 30, 31]. Another important characteristic is that our median survival of ~2.0 years was attained in patients who did not achieve a favorable risk category and therefore unable to achieve the benefits of metastasectomy.

Cost-effectiveness estimates for this study were validated by a health economist with over 30 years of experience (ACG) [29] and were conducted to evaluate the economic impact of MCA in an adjunctive role by considering the added cost for palliation. We acknowledge that thorough cost-effectiveness analyses should include utility estimates for quality-adjusted life years (QALY), as well as sensitivity analyses for both probability and cost assumptions within the framework of a Markov or Monte Carlo decision model [9]. Such in-depth analyses are beyond the scope of this paper, which is primarily focused on the feasibility and survival assessments of MCA in relation to potential cost-effectiveness. Table 4 provides some insight into whether MCA could provide improved cost-effective survival benefits, even when considered as an adjunct to ongoing systemic therapies.

Weaknesses in this study relate to the relatively small patient population of our feasibility and efficacy-based study. Our study sample size was limited to oligo-mCRC patients in order to compare survival outcomes but precluded sufficient analyses of procedural details for pulmonary and/or soft tissue cryoablation. Also, while the definition of oligo-mCRC varies across medical literature, such lesions are generally considered less biologically aggressive and may be more easily controlled [32, 33]. Patients with oligo-mCRC may thus have survival potential greater than traditional stage IV patients. Detailed assessments of progression-free survival were also beyond the scope of this study for local control. As noted, 80% of our patients had some form of additional chemotherapy or targeted therapy which likely also improved our OS estimates. Therefore, any survival gain in our MCA patients may have been simply achieved by selection rather than any MCA effect. Our observed OS was therefore considered adjunctive to systemic regimens when used to calculate LYG and preliminary cost-effectiveness estimates. However, 58% of patients received only BSC following their first MCA procedure, and their observed median survival was similar to those who continued with subsequent systemic therapy (23.5 versus 24.8 months). Moreover the observed survival extension for the chemo-targeted group, though minimal, may be an indication that MCA is able to augment such regimens when used adjunctively. However, with this study encompassing a small patient pool, the similarity in results may be due to less aggressive disease and/or reduced extent of disease in the BSC group. Morbidity associated with chemotargeted regimens may also be a significant factor; however, we do not feel our limited data is able to conclusively make such claims but rather introduce a possible benefit of cryoablation in reducing chemotoxicity. Nevertheless, the future assessment of potential reductions of chemotoxicity by use of MCA, or other ablation modalities, for oligo-mNSCLC appears promising. Further work is needed to convert LYG to QALY for this adjunctive role of MCA, as well as in-depth procedural and periprocedural true cost assessments.

Our cost analysis was also limited. A more comprehensive “social” cost-effectiveness analysis would require enumeration of additional costs on the patient’s end. These would include travel costs (if any) to and from the treatment, foregone wages from lost work days, and any incremental costs incurred by family members in the provision of treatment. Inclusion of these costs would increase the total cost estimates yet would likely not contribute to our already upper bound cost estimates. However, the social and economic impacts of MCA’s very low complication and tumor recurrence rates were also not considered for this study but will likely favor conversion of LYG to QALY, especially with relation to chemotoxicities.

In summary, preliminary estimates of improved survival and cost-effectiveness of cryoablation for the treatment of mCRC provide evidence suggesting the role of cryoablation in metastatic disease should expand, particularly due to low numbers for surgical eligibility. Future potential for reductions in chemotoxicity by utilizing MCA for local control of oligo-mCRC appears promising.

## Figures and Tables

**Table 1:** Patient, procedure, and tumor characteristics. The 6 procedural locations included *lung*, *liver*, and 4 soft tissue sites: *adrenal*, *para-aortic/isolated*, *bone*, *superficial*, and *intraperitoneal. Lung* tumor locations consisted of metastatic lesions in lung parenchyma and/or chest wall but did not include mediastinal or hilar adenopathy. *Superficial* tumor locations consisted of predominantly subcutaneous, muscular, and/or lymph node metastases within the extremities or torso wall. Intraperitoneal tumors were isolated within the abdominal cavity and NOT adherent to bowel. Tumors in *bone* locations were limited metastatic deposits in nonweight bearing locations with the epicenter in osseous structures.

Location	Liver	Lung	Soft tissue				Total*
			*Retroperitoneal*	*Intraperitoneal*	*Bone*	*Superficial*	
Number of patients	48	10	2	2	3	1	59*
Number of procedures	80	21	2	2	5	2	111*
Number of tumors	116	33	2	2	6	2	151
Mean tumor diameter (cm^3^)	3.8	2.7	3.1	3.7	5.6	2.0	3.7
Mean ablation diameter (cm^3^)	5.6	4.6	4.5	6.1	7.4	4.0	5.5

* Totals do not equal the summation because soft tissue is broken down into 4 categories and overlap in the total. Retreatment of a single tumor as well as a single procedure involving multiple locations also overlaps totals.

**Table 2:** Procedure complications. Complication rates per procedure broken down into their respective anatomical locations.

Location	Number of procedures	Grades 1 and 2	Grade 3	Grade 4	Grade 5	Number of complications ≥ grade 3
Liver	80	27	4	3	1	8
Lung	21	7	1			1
Soft tissue						
*Retroperitoneal*	2					
*Superficial*	2					
*Intraperitoneal*	2					
*Bone*	5					

Total*	111	34	5	3	1	9

Total (%)		31%	5%	3%	1%	8%

* Percentage was calculated by using the total number of procedures as the denominator, with overlapped procedures accounted for. Actual procedure number was 111.

**Table 3:** Local tumor recurrence. Total procedural and satellite recurrences broken down by anatomical location of the tumor. Of the 4 observed procedural recurrences, 3 occurred in a single patient on one tumor abutting the hilum. Following additional ablations, a total of 7 (4.6%) recurrences remained, all of which were satellite. Therefore, the overall ablation effectiveness in was 95%.

Location	Number of tumors	Total local recurrences	Procedural (%)	Satellite (%)
Liver	116	11	0%	100%
Lung	33	6	67%	33%
Soft tissue				
*Retroperitoneal*	2	0		
*Intraperitoneal*	2	1	0%	100%
*Bone*	6	0		
*Superficial*	2	0		

Total*	151*	18	4	14

Total%		12%	3%	9%

Total (following reablation)		7	0	7

Total% (following reablation)		5%	0%	5%

* Tumor values overlap in the case of repeat ablations. Actual number of distinct tumors is 151.

**Table 4:** Preliminary cost-effectiveness estimates. Cost-effectiveness estimates for BSC and six established therapies (6–9) for widespread mCRC are noted in conjunction with liberal estimates of cost for MCA. Our proposed adjunctive cost-effectiveness ratio, or ACER, was used to calculate the estimated cost of MCA when paired with systemic regimens.

	BSC	5-FU	5-FU with LV	FOLFOX	FOLFIRI	FOLFIRI + BV	CX and IR	MCA
LYG	0.52	0.71	1.57	1.65	1.66	1.69	0.81	1.97
Total cost ($)**	$4,233	$12,344	$55,793	$94,693	$61,781	$78,245	$37,723	$73,900*
$/LYG	$8,140	$17,386	$35,537	$57,390	$37,217	$46,299	$46,572	$37,513
ACER (Cost/LYG)***	$39,661	$43,779	$65,834	$85,580	$68,874	$77,231	$56,661	Mean: $62,517

* Assumes 1.9 cryoablation procedures per patient and more image intensive followup.

** A conversion factor of 1.67 from pounds to dollars was used to allow easier comparison and conforms to the difference between established definitions of cost efficacy of $100,000 [28].

*** ACER: adjunctive role for, MCA: assumes costs are additive and divided by a total LYG of 1.97 for MCA.

MCA: multisite cryoablation.

5-FU: 5-fluorouracil.

LV: leucovorin.

FOLFOX: 5-FU, leucovorin, and oxaliplatin.

FOLFIRI: 5-FU, leucovorin, and irinotecan.

BV: bevacizumab.

IR: irinotecan.

CX: cetuximab.

## Abbreviations

ACER: Adjunctive cost-effectiveness ratio
BSC: Best supportive care
FOLFIRI: 5-Fluorouracil with irinotecan and leucovorin
FOLFOX: 5-Fluorouracil with oxaliplatin and leucovorin
5-FU: 5-Fluorouracil
LYG: Life-year gained
MCA: Multisite cryoablation
mCRC: Metastatic colorectal cancer
OS: Overall survival
PET: Positron emission tomography
QALY: Quality-adjusted life-year
RFA: Radiofrequency ablation
